# The effect of curcuminoids for treating knee osteoarthritis: A protocol for systematic review and meta analysis

**DOI:** 10.1097/MD.0000000000020556

**Published:** 2020-06-19

**Authors:** Wei Ma, Shilu Wang, Honghao Xu, Wenpeng Xie, Rongxiu Bi

**Affiliations:** a College of Traditional Chinese Medicine, Shandong University of Traditional Chinese Medicine; b Department of Orthopedics, Affiliated Hospital of Shandong University of Traditional Chinese Medicine, Jinan, Shandong Province, China.

**Keywords:** curcuminoids, knee osteoarthritis, protocol, systematic review

## Abstract

**Background:**

Knee osteoarthritis (KOA) is 1 of the commonest cause of disability with joint pain in adults and a burden on healthcare resources. The limitations of current KOA treatment necessitate further researches to discover the more efficacious and safety treatments. There are increasing clinical studies investigating the potential protective effects of Curcuminoids in the alleviation of symptoms in patients suffering from KOA. However, the convincing evidence indicating the efficacy of curcuminoids for patients suffering from KOA remains unclear.

**Methods:**

Several databases including PubMed, Web of Science, Cochrane Library, Embase, Chinese Biomedical Literature Database, Chinese National Knowledge Infrastructure, and Wanfang Database will be searched. And the language was not limited. We will include all Randomized controlled trials that use curcuminoids to treat patients with KOA, regardless of blinding. If the pre-crossover data can be analyzed to avoid carryover effects, the crossover randomized trials also are included. Meanwhile, We will exclude non-randomized controlled trials, qualitative studies, uncontrolled clinical trials and laboratory studies. The primary end point include Western Ontario and McMaster Universities Osteoarthritis Index, visual analog scale scores and Lequesne's pain functional index. The secondary end points are total effective rate and adverse effects. The Review Manager Version 5.3 will be used to perform the data synthesis and subgroup analysis.

**Discussion:**

There are evidences that supports the potential protective effects of Curcuminoids in the alleviation of symptoms in patients suffering from KOA. This systematic review and meta-analysis would provide convincing evidence indicating that curcuminoids relieve the symptoms of patients suffering from KOA.

**Registration:**

Open Science Framework (OSF) registries (https://osf.io/fz29b) with the registration DOI: 10.17605/OSF.IO/FZ29B.

## 1 Introduction

Knee osteoarthritis (KOA) is one of the commonest cause of disability with joint pain in the aging population, that makes significant impairment of physical function.^[1]^ Symptomatic KOA demonstrates the presence of both radiographic KOA and symptoms including pain, stiffness of the joint, crepitation on motion and limitation of motion in the same joint attributable to KOA.^[2]^ KOA has been ranked as the 11th highest contributor to global disability and the 23rd highest cause of disability adjusted life years by the 2017 Global Burden of Disease (GBD) report.^[3]^ Several pharmacological agents have been used for treatment of KOA. Analgesics and non-steroidal anti-inflammatory drugs (NSAIDs) are the current standard of treatment for KOA. Temporary pain relief and hence improvement in physical function may be attained with analgesics but this is not specific to KOA. NSAIDs that are also strongly recommended by AAOS, have some anti-inflammatory and analgesic effects. However, NSAIDs may present severe adverse effects upon prolonged use, it causes a significant GI bleeding risk and are also associated with a variety of renal complications, myocardial infarction and other serious cardiovascular complications.^[4,5]^ Given the above limitations, the more efficacious and safety treatments remain to be discovery. In recent years, medicinal plant extracts had been a surge of interest for KOA treatment, owe identified analgesic, anti-inflammatory and muscle relaxant properties for such therapies.^[6,7]^

Curcuminoids is the primary active component of turmeric, displays a wide range of biological activities including anti-oxidant, anti-inflammatory, and cytotoxicity to numerous cell types.^[8]^ Several vitro studies demonstrated the mechanisms of curcuminoids for KOA treatment. when stimulated with inflammatory IL-1β, lipopolysaccharide or TNF-α, curcuminoids diminished catabolic and degradation action of chondrocyte or cartilage explant models. curcuminoids inhibited the matrix degradation through decreasing the production of MMP-3, MMP-9 and MMP-13 via c-Jun-N-terminal kinases (JNK), nuclear factor kappa-B (NF-κB) and the janus kinase-signal transducer and activator of transcription (JAK/STAT) pathway.^[9,10]^ Curcuminoids stimulated matrix synthesis by restoring glycosaminoglycan (GAG) synthesis and type II collagen.^[11]^ There are increasing clinical studies investigating the therapeutic efficacy of curcuminoids in treating patients with KOA. However, the limitations of these clinical studies is relatively small patient sample size. Accordingly, this study presents a protocol for a systematic review of Curcuminoids in the alleviation of symptoms in patients suffering from KOA.

## 2 Methods

### 2.1 Study registration

This study has been registered in the Open Science Framework (OSF) registries (https://osf.io/fz29b) with the registration DOI: 10.17605/OSF.IO/FZ29B. This protocol adheres to the Preferred Reporting Items for Systematic Reviews and Meta-Analyses Protocols statement guidelines.^[12]^

### 2.2 Objectives

The objective of this study is to evaluate the effect of curcuminoids in the alleviation of symptoms in patients suffering from KOA through updating relative randomized controlled trials (RCTs).

### 2.3 Inclusion criteria for study selection

#### 2.3.1 Types of studies

We will include all RCTs that use curcuminoids to treat patients with KOA, regardless of blinding. If the pre-crossover data can be analyzed to avoid carryover effects, the crossover randomized trials also are included. Meanwhile, We will exclude non-RCT, qualitative studies, uncontrolled clinical trials and laboratory studies. In order to minimize publication bias, the study will not be restricted by language or date of publication.

#### 2.3.2 Types of participants

The patients will be enrolled, who meets following inclusion criteria:

(1) The KOA with mild-to-moderate severity;
(2) bilateral KOA;
(3) age < 80 years.

Diagnosis of KOA will be based on the clinical and radiological criteria defined by the American College of Rheumatology (ACR) and personal report of pain with mild-to-moderate degree on active movement [minimum of 40 mm on a 100-mm visual analog scale (VAS).^[13]^

#### 2.3.3 Types of interventions and comparisons

The interventions of the including RCT will be curcuminoids. The control group will have received placebo and other alternative drugs. We will use subgroup analysis to evaluate the effect of curcuminoids in the alleviation of symptoms in patients suffering from KOA compared with placebo group and alternative drugs drug.

#### 2.3.4 Types of outcome measures

The primary end point:

(1) Western Ontario and McMaster Universities Osteoarthritis Index: Western Ontario and McMaster Universities Osteoarthritis Index subscales consisted of pain (5 items), stiffness (2 items) and physical functioning (17 items). Each item was rated from 0 to 4, totaling scores of 0–20, 0–8 and 0–68 for pain, stiffness and physical functioning subscales, respectively.
(2) VAS scores: VAS was a 100 mm rating scale ranging from ‘no pain at all’ (score 0) to ‘unbearable pain’ (score 100). Patients were instructed to mark a place on the horizontal line of the scale reflecting their knee pain severity.
(3) Lequesne's pain functional index: The pain or discomfort scale has 5 items, the ‘maximum distance walked’ has 1 item, and the functions or activities of daily living (ADL) have 4 items. The pain and ADL scale scores range from 0 (representative of no pain or functional limitation) to 8 (representative of extreme pain or functional limitation). The ‘maximum distance walked’ subscale score ranges from 0 (representative of unlimited) to 6 (representative of less than 100-m walking distance ability). The score is increased by 1 point ‘if the patient uses 1 walking stick or crutch or 2 points if the patient uses 2 walking sticks or crutches.’ Total Lequesne's pain functional index ranges from 0 to 24, with higher scores exhibiting a worse health status

The secondary end points:

(1) total effective rate
(2) adverse effects

### 2.4 Data source and search strategy

#### 2.4.1 Data source

We systematically searched 4 electronic databases PubMed, Web of Science, Cochrane Library, Embase and 3 Chinese literature databases, which are Chinese Biomedical Literature Database, Chinese National Knowledge Infrastructure, and Wanfang Database. And the language was not limited. Our meta-analysis only selected the most recent publication, when the same study had been published in different journals or years. If different studies were done and published by same researchers, all studies ware selected in the meta-analysis.

#### 2.4.2 Search strategy

The main keywords are: curcuminoids, knee osteoarthritis, RCT. Details of search strategy in PubMed as follow:

1 (“Curcuminoids” (combinations of medical subject headings [MeSH] Terms) OR (“curcum” [Title/Abstract]) OR (“curcumin extract” [Title/Abstract]) OR (“curcuma extract” [Title/Abstract])OR (“turmeric” [Title/Abstract]) OR (“turmeric extract” [Title/Abstract]) OR (“curcuminoid” [Title/Abstract])

2 (“knee osteoarthritis” [MeSH Terms]) OR (“arthritis” [Title/Abstract]) OR (“osteoarthritis” [Title/Abstract]) OR (“knee osteoarthritis” [Title/Abstract]) OR (“knee arthritis” [Title/Abstract]) OR (“osteoarthritis of knee joint” [Title/Abstract])

3 (“Randomized, controlled trial” [MeSH Terms]) OR (“Randomized controlled trial∗” [Title/Abstract]) (“clinical study” [Title/Abstract]) OR (“Clinical Trial” [Title/Abstract]) OR (“Controlled study∗” [Title/Abstract]) OR (“Controlled Trial∗” [Title/Abstract])

1 AND 2 AND 3

### 2.5 Data collection

**Figure 1:**
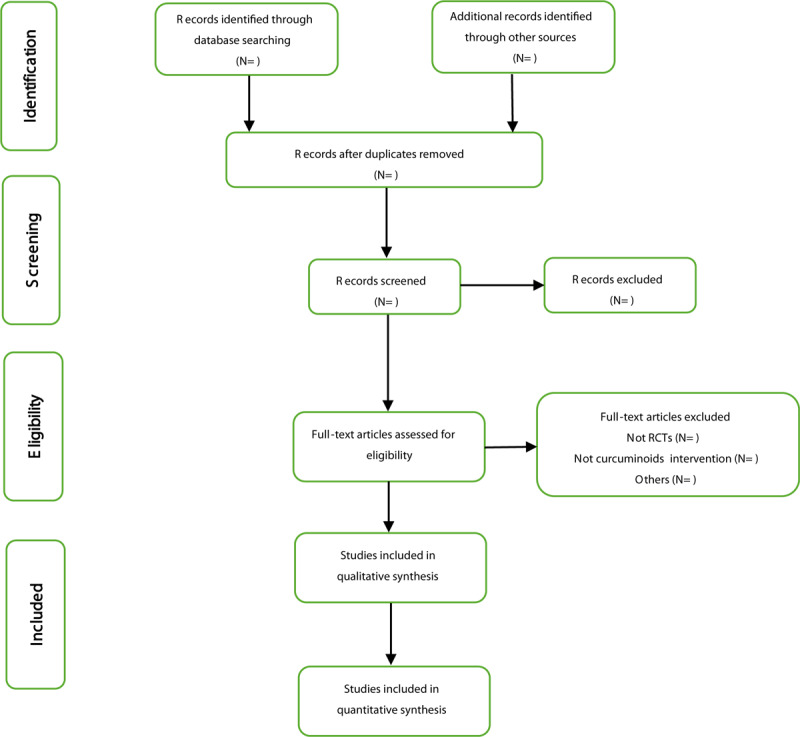
PRISMA flowchart of selection studies. PRISMA = preferred reporting items for systematic reviews and meta-analyses.

Two review authors will search potentially relevant studies and duplicate studies will be deleted and remaining eligible studies will be transferred to EndNote X9 software. Through scanning the titles and abstracts of studies, they will further screen the full-text articles to determine which papers may meet the inclusion criteria. If necessary, the third reviewer was responsible for resolving discrepancies through discussion. Details of study selection will be shown as in a Preferred Reporting Items for Systematic Reviews and Meta-Analyses Protocols flow diagram (Fig. 1).

Relevant data was collected independently by 2 reviewers and the third reviewer was responsible for resolving discrepancies through discussion. Standardized predefined form was used include 4 parts:

(1) basic information: title, the first author, year of publication, and corresponding address;
(2) study characteristics: acupuncture and control intervention, number of sessions, duration, acupuncture points;
(3) the characteristics of participants: sample size, gender distribution, mean age, and race;
(4) results of the study: outcome measures.

When there were missing, errors or ambiguous information in included studies, we will contact original authors of the study. If the author failed to answer the questions, the insufficient information would be neglected or the study would be excluded. Different opinions between reviewers were resolved by communicated with others reviewers.

### 2.6 Sensitivity analysis

We will perform sensitivity analyses to assess the robustness and reliability of results. The methods are changing different methods of analysis (random-effects model or fixed-effect model) and eliminating each of the included studies 1 by 1 and then combine the effect quantities.

### 2.7 Quality assessment

We assessed the quality of involved RCTs by using the tool of Cochrane Collaboration. Two reviewer who achieved date extraction separately evaluated the risk of bias in involved studies. Scoring dissent was decided by the discussion between authors. Several sources of bias consist of Cochrane Collaboration tool, including Random sequence generation, Allocation concealment, Blinding of participants and personnel Blinding of outcome assessment, Incomplete outcome data, Selective reporting, Anything else, ideally prespecified and others. The reviewers would mark every category according to the available information and there are 3 standards, which are high risk (H), low risk (L) and unclear risk (U). H and L risk of bias can be indicated by judging “yes”’ and “no”, when the characterization was not enough to judge the risk of bias, “Unclear” was used. And the study was determined High risk of bias if it received (H) in ≥2 or (U) in ≥3 criteria.

### 2.8 Data synthesis and Statistical analysis

We calculated pooled estimates of risk ratio and weighted mean difference between the intervention and control groups using a random-effects model in the presence of high level of heterogeneity between studies. Fixed effects model was used for meta-analyses of homogeneous data. Between-study heterogeneity was tested by chi-square test (Cochran *Q* test) and I2. Heterogeneity was considered low if *I*^2^ < 30%, moderate if *I*^2^ = 30% to 75%, and high if *I*^2^ > 75%. Chi-square *P* < .1 was set as a level of significant heterogeneity. Constructing a Funnel Graph was used to detect publication bias. All the statistical analyses were performed using Cochrane Program Review Manager Version 5.3 (Cochrane Collaboration, Oxford).

### 2.9 Ethics

This study was approved by the Shandong University of Traditional Chinese Medicine ethics committee. And all subjects were asked to provide written consent.

## 3 Discussion

The protective effects of curcuminoids for treating KOA has multiple molecular mechanism. Previous studies showed reactive oxygen species can impair intra-articular segments and components of joints such as proteins, lipids and nucleic acids.^[14]^ Reactive oxygen species can promote chondrocyte apoptosis, MMP expression and production of mediators involved in pain and disturb cartilage matrix homeostasis.^[15]^ Curcuminoids are potent antioxidants and have been shown to modulate oxidative stress through various mechanisms, an effect that leads to reduced lipid peroxidation and attenuation of oxidative damage to DNA and proteins. Above all, curcumin has the ability to inhibit inflammatory cell proliferation, invasion, and angiogenesis through multiple molecular targets and mechanisms of action.^[16]^ Curcumin inhibited the NF-kappaB targets including COX-2 and MMP-9, indicate that it has nutritional potential as a naturally occurring anti-inflammatory agent for treating OA through suppression of NF-kappaB mediated IL-1beta/TNF-alpha catabolic signalling pathways in chondrocytes.^[9]^ Curcuminoids has been identified that it can also effectively decrease the expression of pro-inflammatory cytokines such as macrophage chemotactic protein-1, IL-1β, IL-6, tumor necrosis factor-α and prostaglandin E2.^[17]^ Curcuminoids also has been indicated that increase chondrocyte survival through down-regulation of inflammation-induced apoptosis.^[18]^

There are evidences that supports the potential protective effects of Curcuminoids in the alleviation of symptoms in patients suffering from KOA.^[19,20]^ Several lines of clinical studies confirmed that curcumin is effective and safe for treating KOA. In particularly, curcumin have been an anti-inflammatory agent, extensively used in traditional medicine. However, some previous published studies always have the several limitations including the number of patients was relatively small and incomplete demographic data. This systematic review and meta-analysis would provide convincing evidence indicating that curcuminoids relieve the symptoms of patients suffering from KOA.

## Author contributions

**Conceptualization:** Wei Ma, Shilu Wang

**Funding acquisition:** Rongxiu Bi

**Methodology:** Wenpeng Xie, Honghao Xu

**Project administration:** Wei Ma, Shilu Wang

**Writing – original draft:** Wei Ma, Shilu Wang

**Writing – review & editing:** Wei Ma, Shilu Wang
